# Functional implant positioning in total hip arthroplasty and the role of robotic-arm assistance

**DOI:** 10.1007/s00264-022-05646-0

**Published:** 2022-12-11

**Authors:** Andreas Fontalis, Rhody David Raj, Woo Jae Kim, Ayman Gabr, Fabrice Glod, Constant Foissey, Babar Kayani, Pierre Putzeys, Fares S. Haddad

**Affiliations:** 1 Department of Trauma and Orthopaedic Surgery, University College Hospital, London, UK; 2 Division of Surgery and Interventional Science, University College London, Gower Street, London, WC1E 6BT UK; 3 Hôpitaux Robert Schuman, Luxembourg City, Luxembourg; 4 Department of Orthopedic Surgery and Sport Medicine, Croix-Rousse Hospital, FIFA Medical Center of Excellence, Lyon, France

**Keywords:** Biomechanics, Total hip arthroplasty, Robotic-arm assistance, Spinopelvic mobility, Functional implant positioning, Impingement, Centre of rotation

## Abstract

**Introduction:**

Accurate implant positioning, tailored to the phenotype and unique biomechanics of each patient is the single most important objective in achieving stability in THA and maximise range of motion. The spine-pelvis-hip construct functions as a single unit adapting to postural changes. It is widely accepted in the literature that no universaltarget exists and variations in spinopelvic mobility mandate adjustments to the surgical plan; thus bringing to the fore the concept of personalised, functional component positioning.

**Methods:**

This manuscript aims to outline the challenges posed by spinopelvic imbalance and present a reproducible, stepwise approach to achieve functional-component positioning. We also present the one-year functional outcomes and Patient Reported Outcome Measures of a prospective cohort operated with this technique.

**Results and Conclusion:**

Robotic-arm assisted Total Hip Arthroplasty has facilitated enhanced planning based on the patient’s phenotype and evidence suggests it results in more reproducible and accurate implant positioning. Preservation of offset, avoiding leg-length discrepancy, accurate restoration of the centre of rotation and accomplishing the combinedversion target are very important parameters in Total Hip Arthroplasty that affect post-operative implant longevity, patient satisfaction and clinical outcomes.

## Introduction

As the number of primary cases performed annually is steadily rising, 100,000 in the UK alone, total hip arthroplasty (THA) is the cornerstone surgical management for osteoarthritis. Having been widely employed by arthroplasty surgeons for over 60 years, novel developments are imperative to solve the challenges that have emerged over the decades. Prosthetic hip dislocation is a frequent complication after THA with a risk of instability of 0.17 to 1.74% within two years [1], resulting in the most common need for revision surgery, a poorer quality of life and higher medical costs [2–4]. Several operative factors contribute to hip stability, such as preservation of the dynamic and static hip stabilisers and restoration of joint biomechanics; however, the pivotal contribution to hip stability is accurate acetabular cup positioning. Consensus of the recent literature shows that there is no common optimal cup position for all patients due to variations in spinopelvic anatomy. Advances in surgical technology have permitted the assessment of these variations and have facilitated the provision of a surgical plan to achieve optimal implant positioning through the use of robotic arm assistance [5].

### Spinopelvic motion

Spinopelvic motion is a complex chain of movements between the spine, pelvis and hips which accommodates for postural change [6]. As we transition from a standing to sitting position, the sacrum moves posteriorly and there is loss of lumbar lordosis which increases acetabular anteversion [7]. A combination of movements helps achieve this and the reported values of normal spinopelvic movement when sitting are as follows; 55–70° of hip flexion, 20° of posterior pelvic tilt and 20° loss of lumbar lordosis [8]. A stiff lumbar spine impacts this kinematic chain and range of movement of the pelvis and as a result reduces the posterior tilt and functional anteversion of the acetabulum [9]. Hence, patients compensate with hyperflexion of the hip which increases the risk of impingement [8].

### Spinopelvic parameters

Anterior pelvic plane (APP) is defined as the plane between the pubic symphysis and the anterior superior iliac spines (ASIS). The anterior pelvic plane tilt (APPt) is the angle between a vertical coronal plane and the APP [10].

Pelvic incidence (PI) is an anatomical parameter of a constant value unique to each individual and is independent of the position of the pelvis. It is defined as the angle between the perpendicular line to the S1 end plate at its midpoint and the line connecting this point to the centre of the femoral head [11]. The pelvic incidence is an algebraic sum of the sacral slope and pelvic tilt [12].

**Fig. 1:**
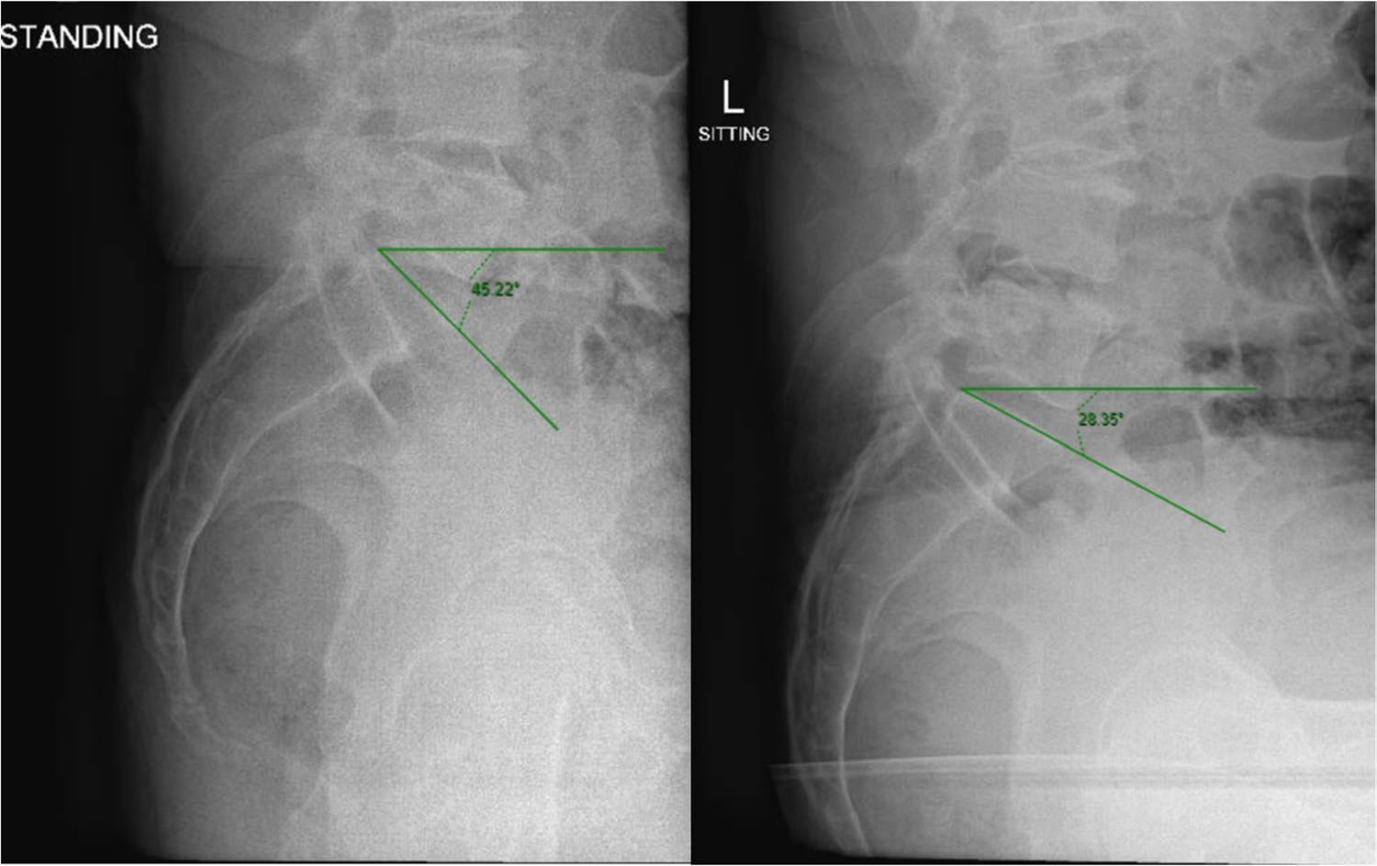
Lateral pelvis plain radiographs demonstrating sacral slope measurements in standing (left) and sitting (right) positions

Sacral slope (SS) is defined as the angle between the tangential line at the upper end of the S1 end plate and the horizontal line [13]. It is a dynamic parameter which allows us to quantify and assess spinopelvic movement through postural changes. An accurate measurement of spinopelvic mobility is the difference in sacral slope angle between sitting and standing (ΔSS) (Fig. 1).

Lumbar lordotic angle is the angle subtended by the two lines drawn from the superior endplate of L1 and S1. When PI minus LL is > 10°, this is known as a flatback spinal deformity [14].

### Classification

An accurate method of assessing dynamic changes in spinopelvic mobility is the change in sacral slope (ΔSS). Stefl et al. have established that the normal range of (ΔSS) is between 10 and 29° throughout the motion of sitting and standing and described five patterns of spinopelvic mobility: normal, hypermobile, ‘stuck standing’, ‘stuck sitting’ and kyphotic [15]. A ΔSS of greater than 30° has been classified as hypermobility but can be a normal variant for certain patient groups, namely, women and younger patients. It has been linked with a protective advantage against the risk of impingement as there is less range of movement required at the hip whilst sitting [16]. However, when there is excessive spinopelvic movement due to lumbar kyphosis whilst in the sitting position, this is considered unbalanced hypermobility. This is commonly associated with stiff hip flexion, ≤ 50° of flexion and patients with a BMI in excess of 40 resulting in a posterior pelvic tilt when sitting due to their large body habitus [16].

On the contrary, a ΔSS of less than 10° signifies a stiff spinopelvic unit and is predominantly due to degenerative disease of the lower spine or spinal fusion. As a result, there is less pelvic extension and subsequently less acetabular anteversion when transitioning into a sitting position. It is stated that for each degree loss of pelvic extension, there is a loss of 0.8° of acetabular anteversion [17]. Stiffness in the spinopelvic unit can be further delineated by the position of the pelvis. A ‘stuck standing’ pelvis is whereby the absolute sacral slope value is > 30° whilst sitting and the pelvis maintains an anterior tilt [15]. Therefore, patients compensate by increasing flexion at the hip joint which increases the risk of anterior impingement and posterior dislocation when seated [17]. The other pattern of stiffness is termed ‘stuck sitting’ whereby the pelvis is fixed in a posteriorly tilted position and does not tilt anteriorly whilst standing and maintains an absolute sacral tilt value < 30° [15, 16]. As a result, there is an increased risk of posterior impingement in hip extension when standing [17].

Vigdorchik et al. have proposed a hip-spine classification based on spinal deformity and stiffness. The alignment of the spine is determined by the pelvic incidence minus lumbar lordotic angle, and patients are divided into normal alignment (PI-LL < 10) or flatback deformity (PI-LL > 10). Patients are then further subdivided with regards to the mobility of their spine; patients with a ΔSS > 10 are considered normal, whereas those with a ΔSS < 10 are considered stiff. Thus, Vigdorchik has proposed four categories of patients: 1A, normal spinal alignment and mobility; 1B, normal spinal alignment but stiff spine; 2A, flatback deformity with normal mobility; 2B, flatback deformity with stiffness [18].

### Functional cup positioning

Functional cup position is a term which has recently gained popularity with the notion that previously described safe zones by Lewinnek and Callanan does not necessarily ensure that patients will not dislocate following their total hip arthroplasties. Evidence has emerged that despite optimal cup positions within the aforementioned safe zones, dislocations continue to occur. Abdel et al. performed a retrospective cohort study which found that 58% of the dislocating hips had the acetabular cup within Lewinnek’s safe zone [19]. Furthermore, a systematic review by Seagrave et al. found that seven of the 11 studies which compared dislocation rates with combined anteversion, and inclination revealed a greater proportion of dislocations occurred within Lewinnek safe zone [20].

The effects of spinal stiffness on the outcomes of THA and dislocation rates have been demonstrated in several studies. A large retrospective study by Buckland et al. has reported a statistically significant higher rate of dislocation of 2.96 and 4.12% for those who underwent a THA with one to two level fusions and three to seven level fusions, respectively, in comparison to 1.55% in those who have not undergone any previous spinal fusions [21]. Similar findings have been echoed by a large retrospective study which concluded that those who undergo THA having previously undergone lumbosacral fusions (LSF) have a 46, 60 and 106% greater risk of dislocating in comparison to those who undergo LSF one year, two years and five years post-THA, respectively [22]. In addition, a recent systematic review and meta-analysis which assessed six studies investigating a total of 1,456,898 THA patients found that there was a higher rate of dislocation and revisions in patients with previous lumbar fusions than those without [23].

Thus, the concept of a patient-specific cup implant position is gaining traction, which considers spinopelvic alignment and mobility of their spine to help dictate the individual’s safe zone. Acetabular component position recommendations have been developed by Luthringer et al. and Vigdorchik et al. based on the Hip Spine Classification [14, 24]. Patients with normal alignment and stiff spine (1B) should have the acetabular component placed at 30° of anteversion on a standing AP pelvis view in order to prevent anterior impingement and posterior dislocations. In patients with a flatback spinal deformity but normal mobility (2A), the target anteversion should be 25–30° to the functional pelvic plane of the body rather than the APP. The spinal deformity causes this subset of patients to stand with a posterior pelvic tilt and hence there is increased functional acetabular anteversion when standing. There is a risk of anterior dislocation if the version of the cup is measured against the APP which will be posteriorly tilted in these patients rather than the functional pelvic plane. The patients with the highest risk of dislocations are those with a stiff flatback deformed spine (2B) and it has been recommended to target 30° of anteversion to the functional pelvic plane on the standing AP pelvis to prevent posterior dislocations [14].

There is yet to be compelling evidence to link restoration of femoral offset with improved stability for THA; however, theoretically, it should facilitate greater range of movement before impingement. A large retrospective analysis by Vigdorchik et al. demonstrated that from their cohort of 12,365 patients, there were a total of 51 dislocations, of which 96% had a standard-offset implant [25]. Furthermore, the authors utilised a novel programme to model range of movement between standard and high offset stems and found each 1 mm of offset increased flexion by 5° and 4° of extension before impingement [25].

### The role of robotic-armassisted surgery

Once optimal acetabular cup positioning has been determined pre-operatively based on patient-specific anatomy and spinopelvic stiffness, implantation must be executed with great precision; otherwise, the planning would be rendered futile. To fulfil this need, advances in surgical technology have led to the assistance of robotic devices in the operating theatre to achieve enhanced surgical planning and attain precision in bony cuts and prostheses implantation. The first robots in THA were fully active, where the robot would independently perform the cuts and implant the components based on the pre-operative plan and the surgeon would monitor, assist and intervene if needed. Less than satisfactory outcomes emerged, revealing an unexpectedly higher dislocation rate compared to manual THA as well as a higher rate of revision surgery, more frequent soft-tissue complications and an 18% conversion to manual THA [26, 27]. This sparked the advent of the second generation of semi-active robots, where the surgeon performs the operation with the assistance of a robotic arm providing a haptic feedback and restriction within a pre-programmed spatial window when making the bone cuts. The industry offers a range of semi-active systems with the MAKO Robotic arm Interactive Orthopaedic (RIO) system (Stryker, USA) being the most commonly used [28]. The latest software version (MAKO 4.0) has introduced ‘virtual range of motion’, a tool that offers real-time feedback, allowing the surgeon to visualise the effect of altering implant position as well as being able to visualise impingement. Studies assessing the outcomes of semi-active robots have shown positive results, such as better-positioned acetabular cups, improved functional outcomes, reduced post-operative pain and a short learning curve [29–31].

The current literature is suggesting that there is no universal fit that can be applied to all patients. Arthroplasty surgeons should be advancing towards a patient-specific approach to reduce dislocation rates by tailoring cup positioning according to patient anatomy, which is enabled by developments in surgical technology. This study aims to delineate the challenges posed by spinopelvic imbalance and present a pragmatic and reproducible workflow utilising robotic arm assistance to achieve functional cup positioning. Furthermore, we present the one year results of a prospective patient cohort undergoing RO THA with this technique.

### Case presentation

**Fig. 2:**
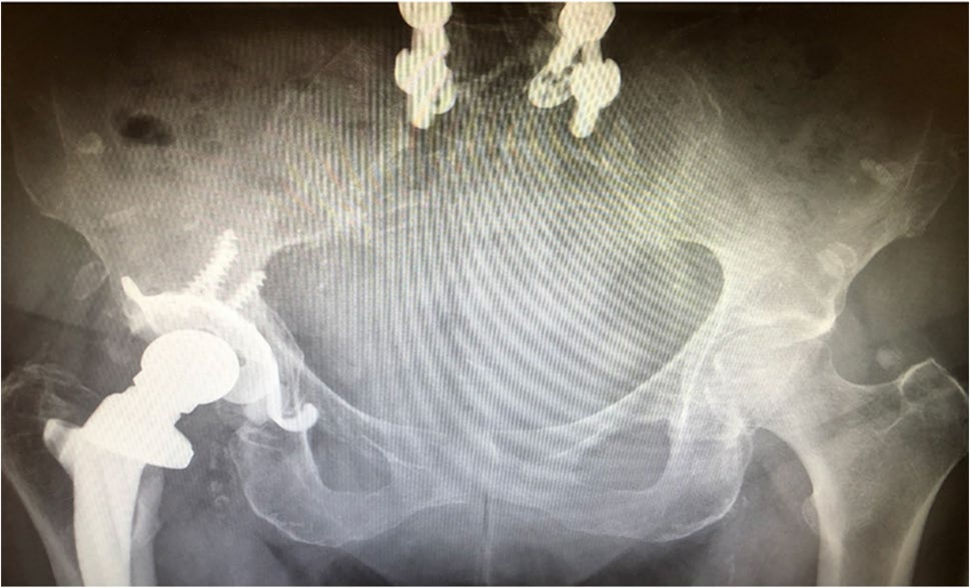
AP pelvis radiograph showing previous right THA, the arthritic changes in the left hip and spinal fusion metalwork

This case presentation demonstrates the preo-perative planning and intra-operative decision-making to achieve functional component positioning. The presented case concerns a 76-year-old female with left hip advanced osteoarthritis and a previous history of multiple spinal surgeries and single-level spinal fusion (Fig. 2).

**Fig. 3:**
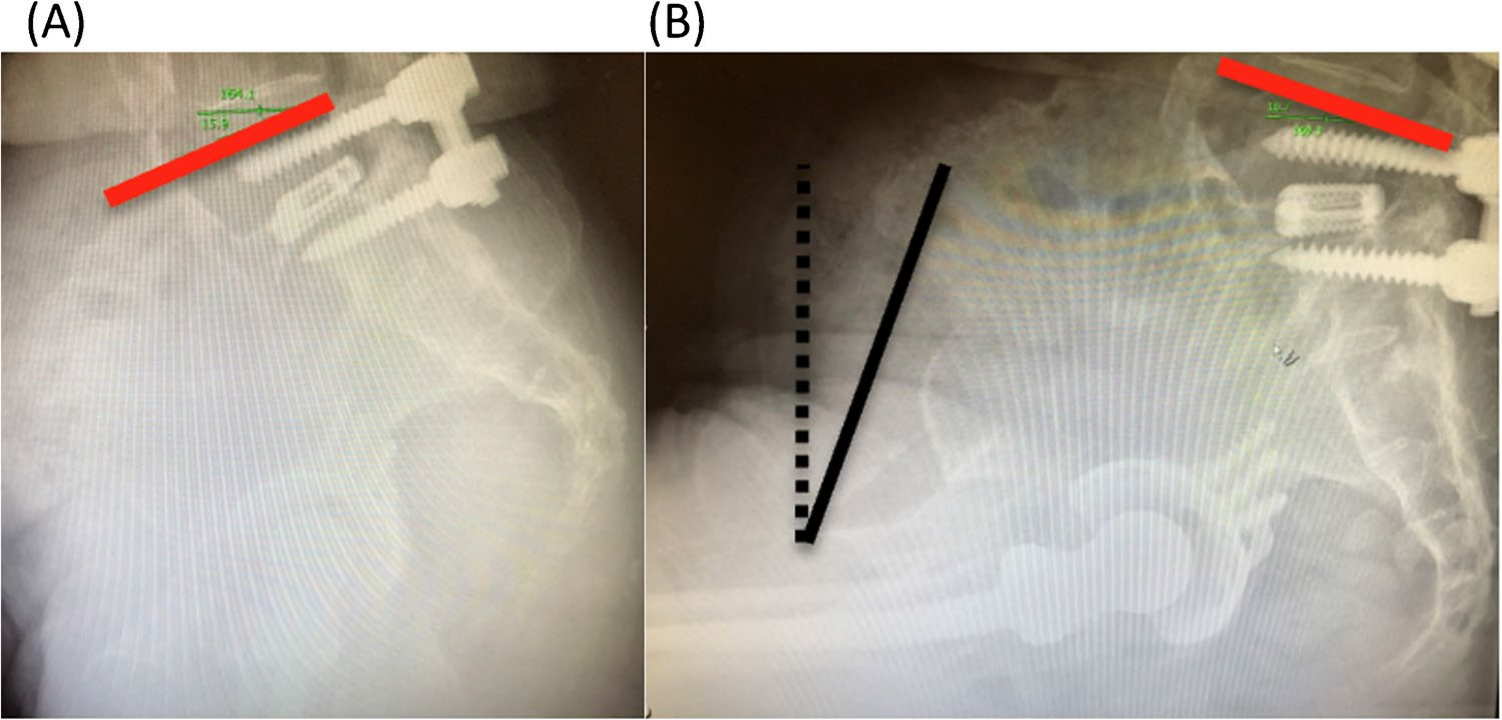
Sacral slope (SS) measurement: **A** standing and **B** sitting positions

Pre-operative radiographs showed that her sitting and standing SS were − 11 and 20°, respectively (Fig. 3). The ΔSS was 31° indicating a hypermobile spinopelvic unit according to the Stefl classification [15] with a normal spinal alignment and mobility according to the international hip spine classification [18]. These measures were entered on the robotic software to aid into the 3D planning of the case.

**Fig. 4:**
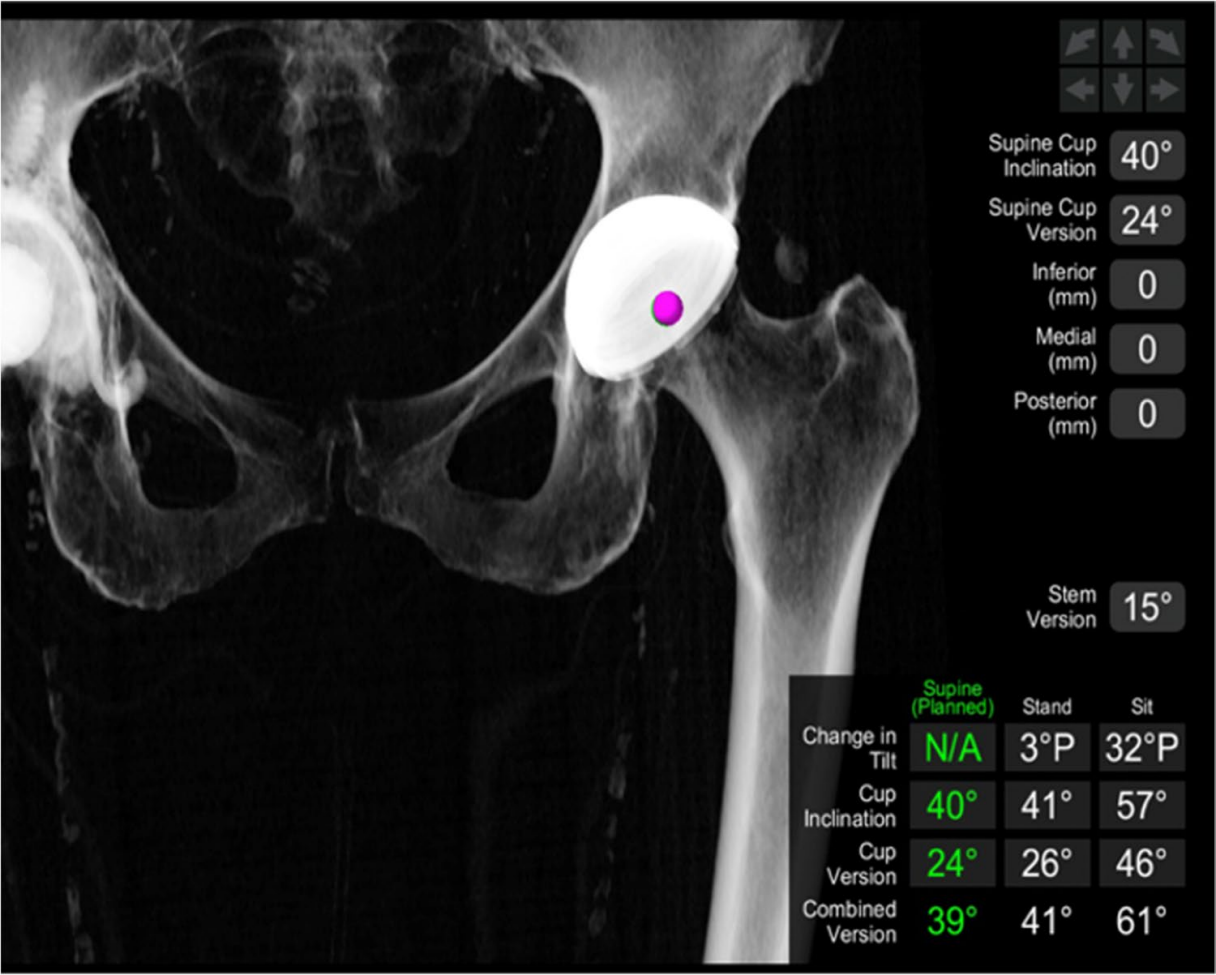
Preo-perative planning with anticipated changes to cup inclination and version in both sitting and standing positions

**Fig. 5:**
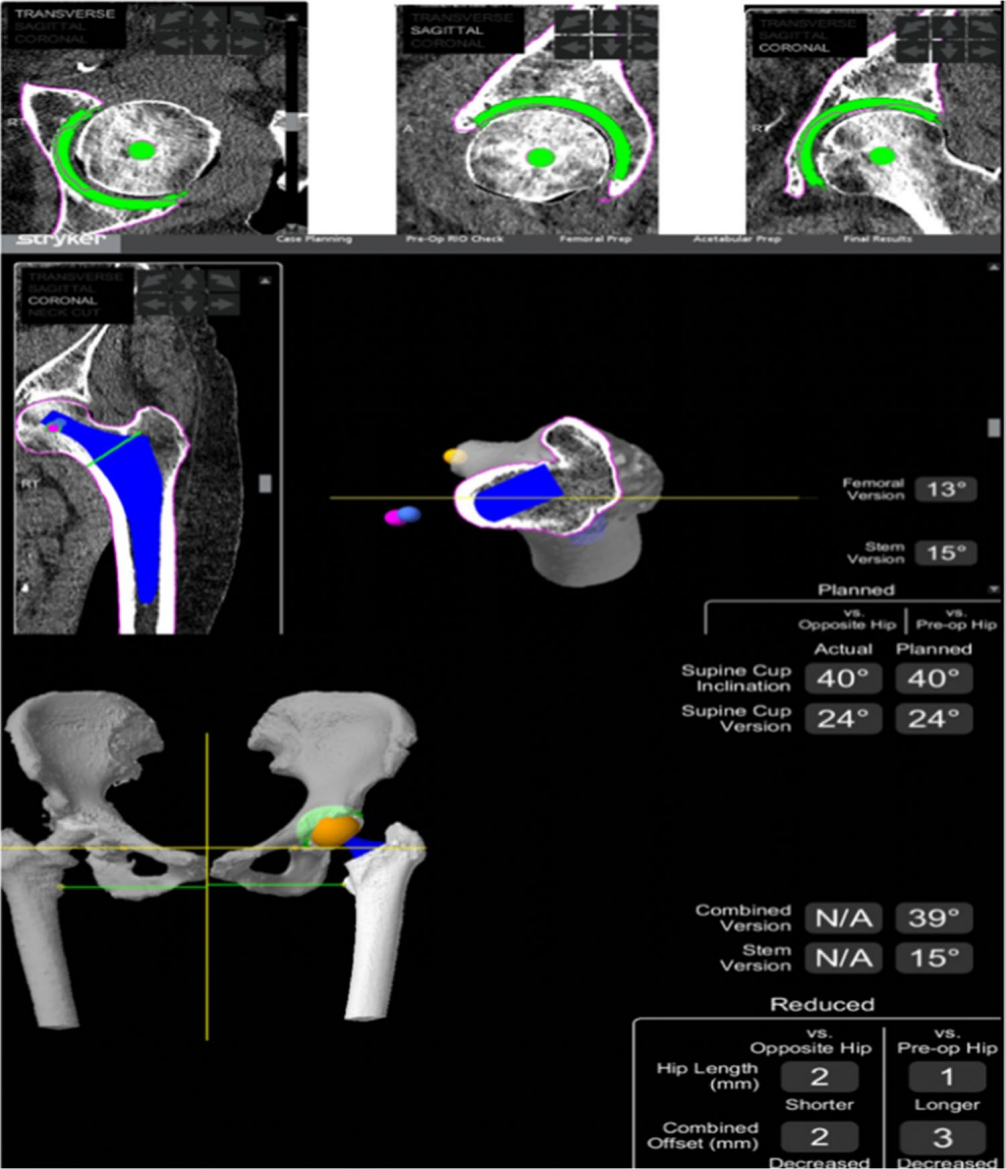
Pre-operative planning demonstrating combined version and expected difference in hip length and offset compared to the opposite hip and pre-operative hip

The pre-operative 3D planning is demonstrated in Fig. 4. The starting point for our cup inclination and version in the supine position were 40 and 20°, respectively; however, owing to the individual anatomy, spinopelvic motion and virtual ROM performed, this was changed to 24° of anteversion to achieve optimal stability. The software shows the changes to the cup inclination and version in both sitting and standing position (Fig. 4). The native stem version was 13°, and the planned stem version was 15°. This allowed a planned combined version of 39° (Fig. 5).

The next step was to check the virtual range of movement (VROM). The robotic software allows to examine the planned hip replacement through maximum range of flexion and extension to identify any potential impingement at the bone-on-bone, bone-on-implant or implant-on-implant interfaces. This feature enables to virtually identify dynamic impingement and acquire instant feedback of adjusting or changing component positioning.

**Fig. 6:**
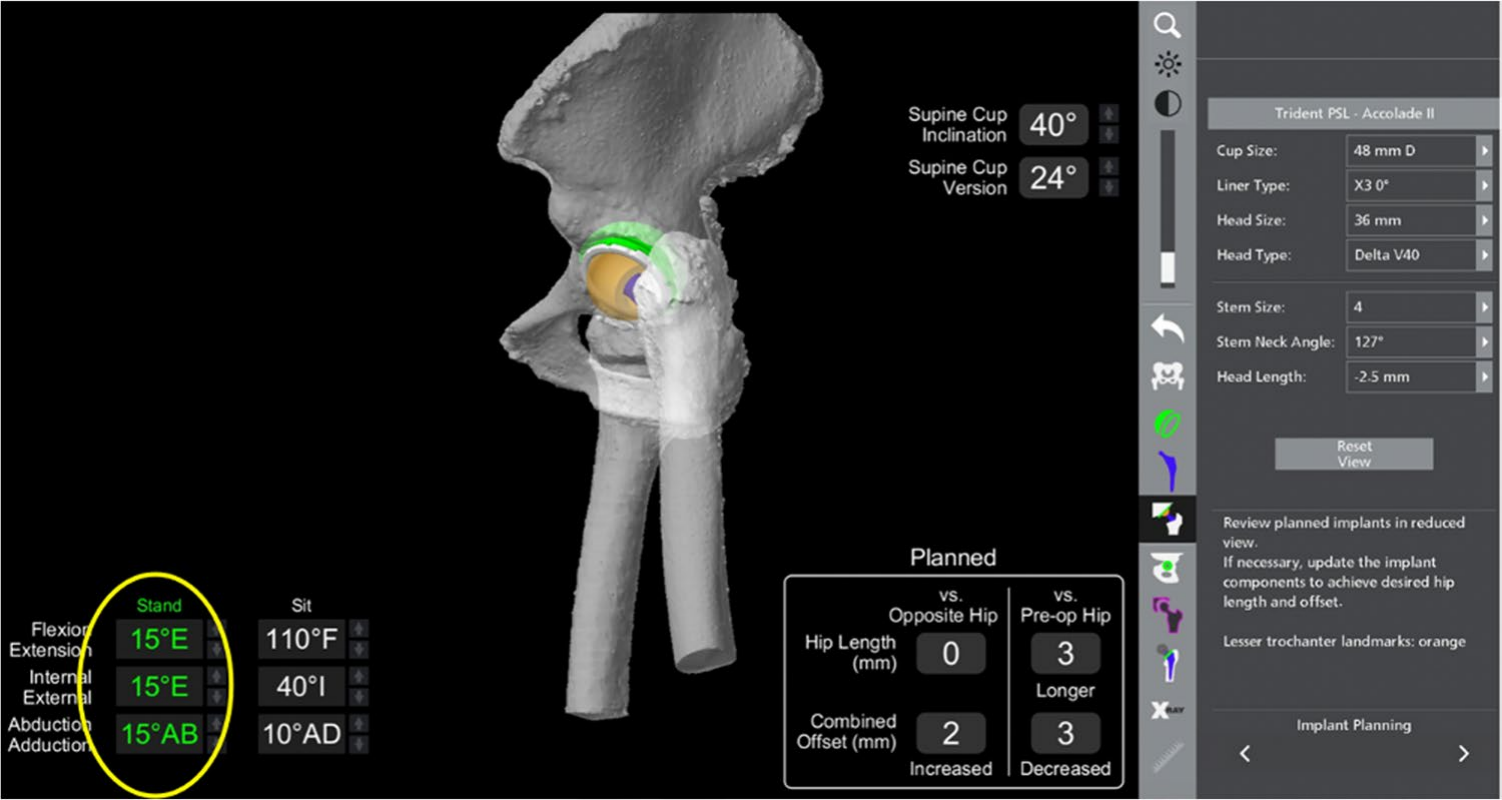
Virtual Range of Motion (VROM) in hip extension with no impingement

In this case, the VROM showed no impingement in standing position with the hip at 15° of extension, 15° of external rotation and 15° of abduction (Fig. 6).

**Fig. 7:**
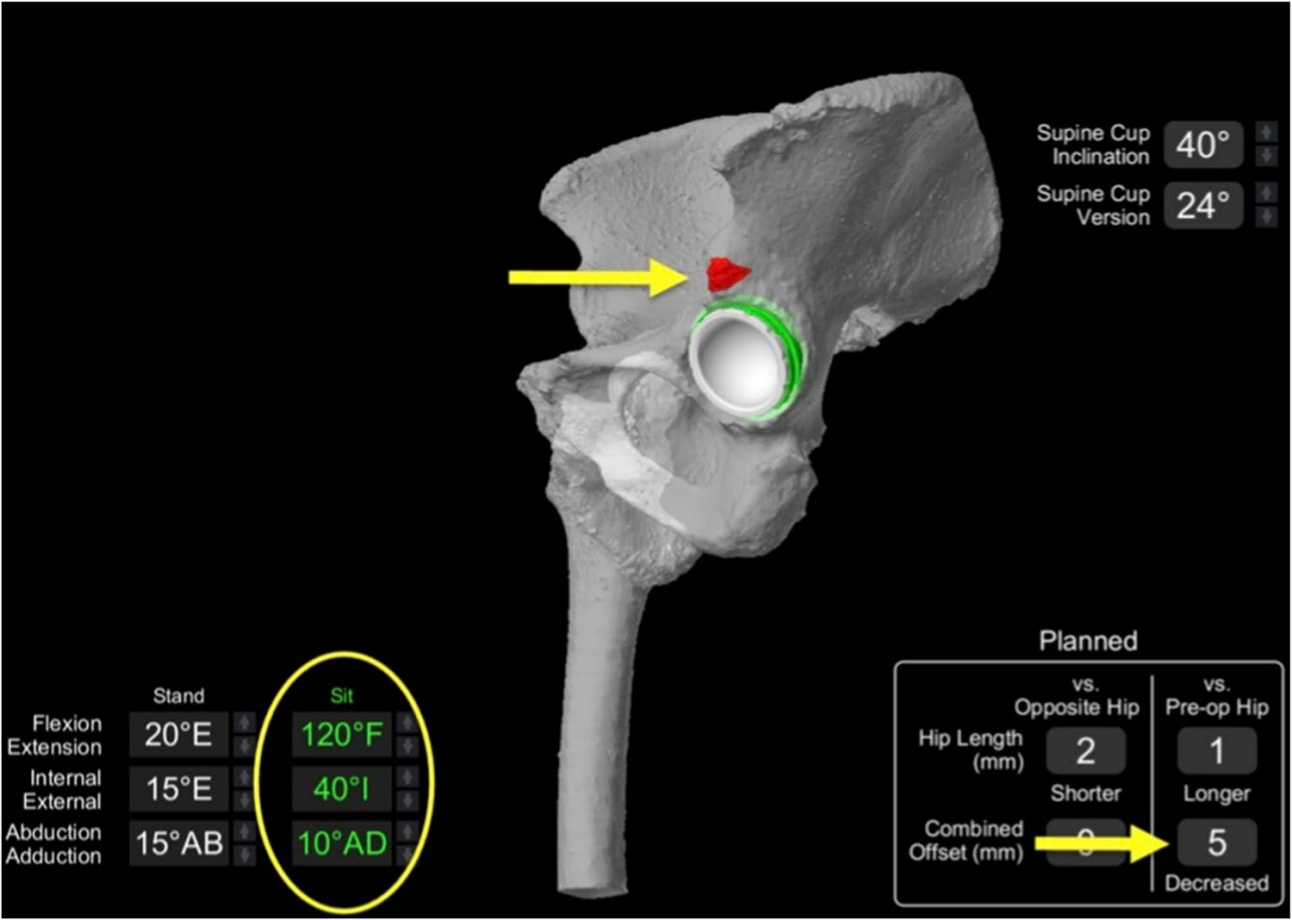
VROM in deep hip flexion and internal rotation demonstrating bone-on-bone impingement (red mark)

However, bone-on-bone impingement was noted at 120° of hip flexion, 40° of internal rotation and 10° of adduction (Fig. 7). Using the robotic software, the combined offset was then increased using a + 5-mm femoral head.

**Fig. 8:**
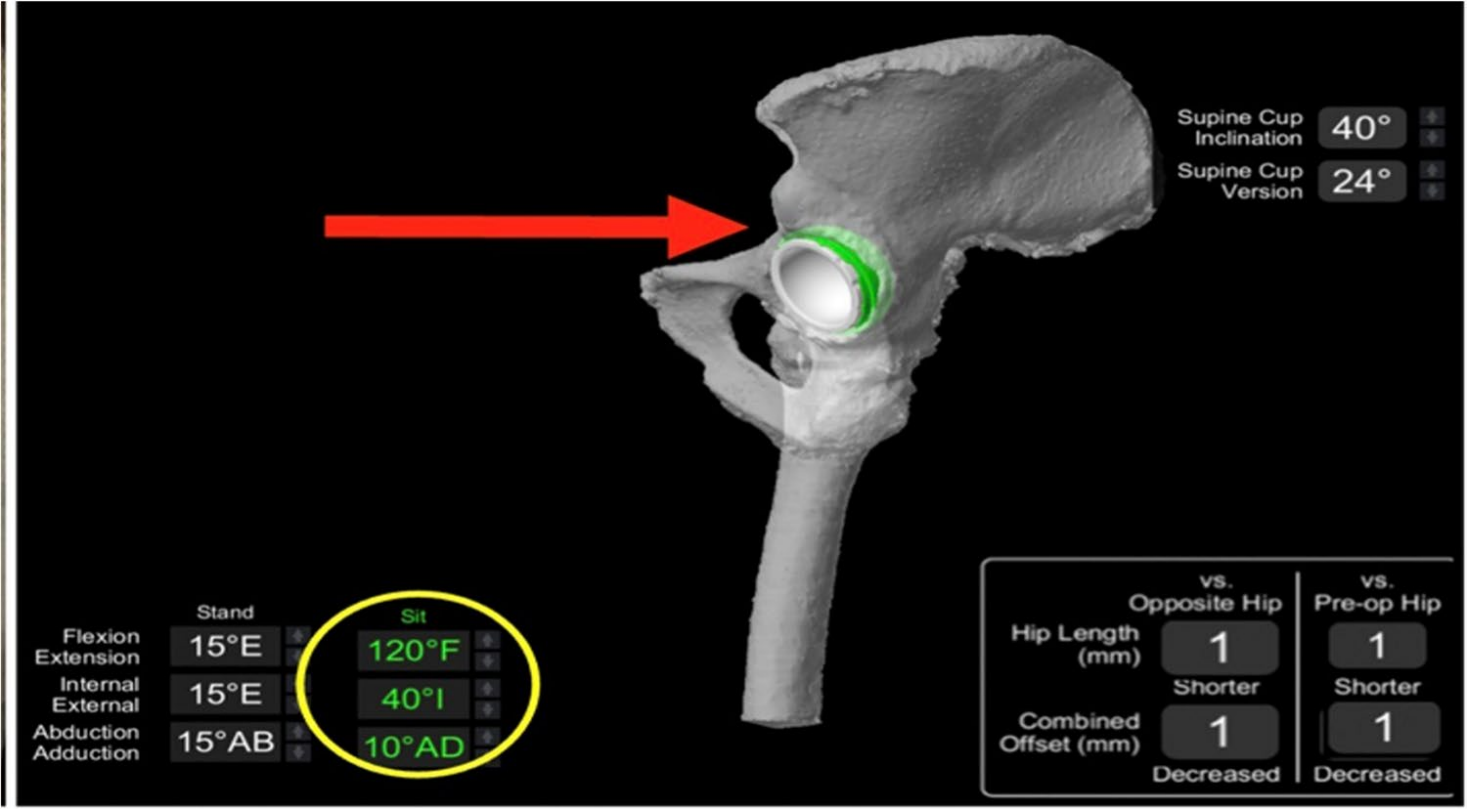
VROM checked after increasing the offset with + 5 mm femoral head that resulted in elimination of impingement in deep flexion

The VROM was rechecked again to confirm impingement was eliminated following the offset increase (Fig. 8).

## Materials and methods

### Functional, radiological and patient-reported outcome measures utilising functional component positioning in THA

#### Study design and participants

This prospective cohort study included patients undergoing primary robotic arm–assisted THA for symptomatic hip osteoarthritis. All operations were performed in a single unit and ethical approval was obtained from the institutional review board (Reference No: CIV-LU-21–09-037787). Written consent was obtained from all research participants.

All procedures were performed using the posterior approach by a senior consultant surgeon with extensive experience in robotic arm–assisted THA.

Radiological parameters pertaining to the orientation of the implanted cup, stem version and combined anteversion were recorded. The Oxford Hip Score (OHS), Hip Disability and Osteoarthritis Outcome Score (HOOS) and Forgotten Joint Score (FJS) were collected pre-operatively and at two years.

#### Pre-operative functional planning

Three-dimensional reconstructions were used based on CT pelvis scans to create a functional, patient-tailored plan using the MAKO total hip system (Stryker Corp, Mako Surgical Corp, Ft. Lauderdale, FL). The following workflow was utilised to formulate a patient specific plan: First, the spinopelvic measurements obtained from sitting and standing lumbar spine lateral radiographs were imported into the robotic software. The starting point for acetabular cup orientation was 40° inclination and 20° anteversion. The cup position was subsequently evaluated in the coronal, sagittal and transverse plane to ensure sufficient bony coverage, restoration of the centre of rotation and to ascertain the posterior wall.

In relation to stem positioning, the two-dimensional predicted post-operative appearance that is available with the robotic software was evaluated to ensure the appropriate stem size, offset and correct any varus or valgus malalignment. Following this, the desired stem anteversion was chosen based on the transverse and sagittal views, taking into account the native version and combined anteversion.

The latest robotic software has introduced an innovative feature, the virtual Range of Motion (vROM) tool, allowing real-time assessment of impingement and ROM. Our testing position in flexion was 110° flexion and 40° internal rotation, whereas in extension the hip was tested at 10° extension and 15° external rotation. On the basis of the site and type of impingement, a functional plan was established adjusting the medio-lateral position of the cup, changing the orientation, offset or size.

#### Surgical technique and implants

The enhanced workflow was used in all cases providing intra-operative feedback regarding broach and stem version, combined anteversion and guiding neck resection. Robotic arm assistance was used to perform reaming in the desired plane with the utilisation of stereotactic boundaries. The acetabular cup was also positioned with the assistance of the robotic arm and the haptic tunnel that maintains the planned orientation during implantation. In this prospective cohort study, all patients received a cementless femoral stem (Accolade II; Stryker, Mahwah, NJ, USA) and a peripheral self-locking porous acetabular shell (Trident PSL shell; Stryker).

### Statistical analysis

Descriptive statistics are presented for all qualitative data. Skewness, kurtosis and boxplots as well as statistical tests including the Shapiro–Wilk and Kolmogorov–Smirnov tests employed to assess whether the normality assumptions were violated. All analyses were performed using the IBM SPSS statistics 162 software for Mac, version 28 (IBM Corp. Released 2021. Armonk, NY: IBM Corp.).

## Results

Our prospective cohort study included 30 participants undergoing robotic arm–assisted THA with functional cup positioning. The mean age of the patients was 69.5 years (SD 9.5) with an equal number of males and females. At baseline, the mean change in sacral slope (ΔSS) between sitting and standing for the entire cohort was 23.4 ± 12.8, while the mean pelvic incidence was 55 ± 13.

**Fig. 9:**
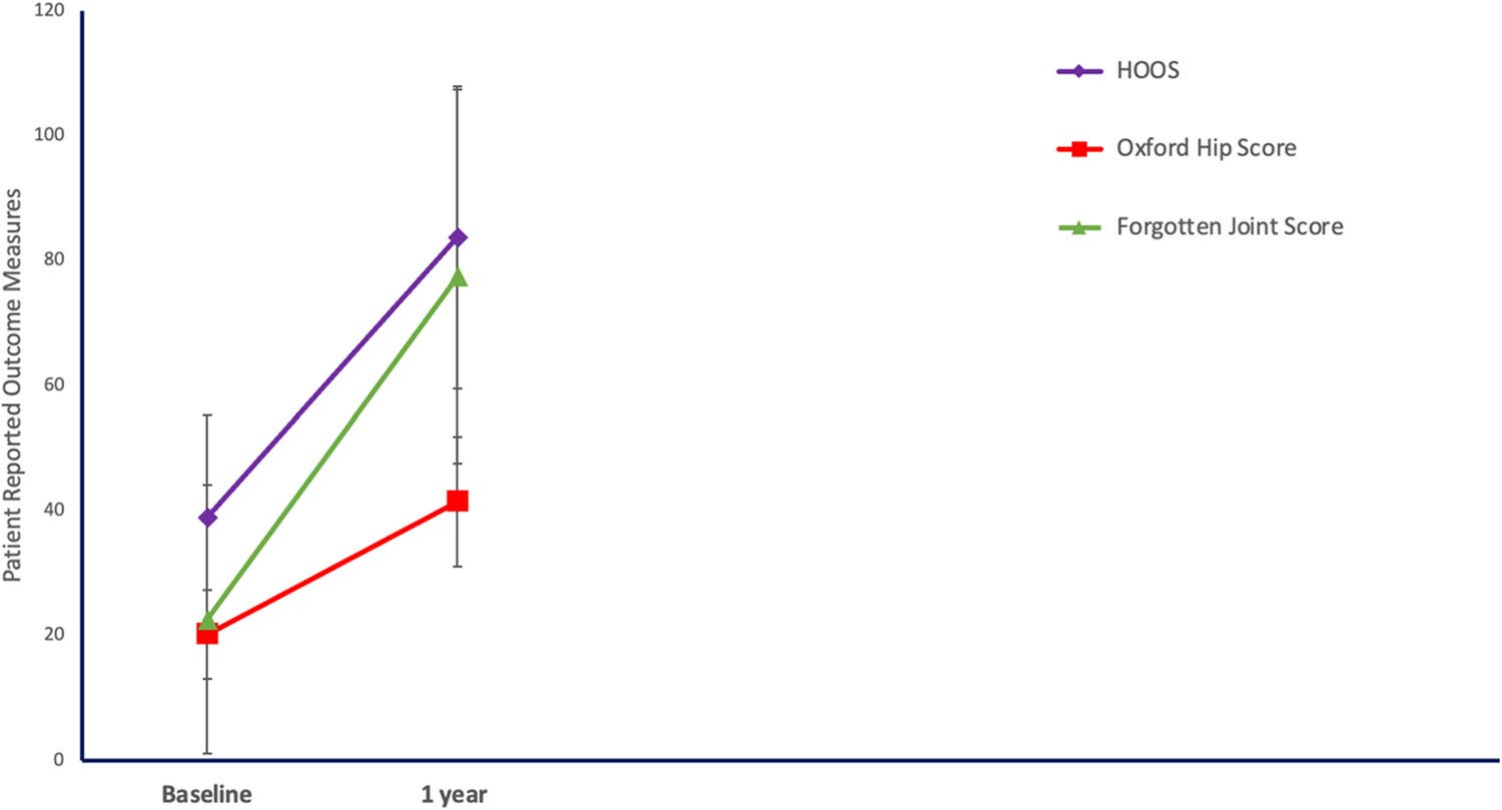
Graph representing PROM scores at baseline and 1 year post-operatively

Baseline PROMs and ROM are presented in Table 1; mean HOOS score was 39 ± 16.35, mean OHS 20.3 ± 7.1 and median FJS 14.6 (IQR 8.3 to 27.3). Table 2 and Fig. 9 illustrate PROM scores and functional outcomes at 1 year’s follow-up. All PROMs improved significantly from baseline and patients experienced a significant improvement in ROM (Table 2).

**Table 1:** Baseline patient-reported outcome measures and range of motion amongst patients undergoing robotic arm–assisted THA with functional cup positioning

Variables	Robotic arm–assisted THA (*N* = 30)
HOOS (%) HOOS pain (%) HOOS symptoms (%) HOOS activity of daily living (%) HOOS sport and recreation function (%) HOOS hip-related quality of life (%)	39 ± 16.35 43.12 ± 19.8 41.25 ± 15.48 42.15 ± 17.72 26.58 ± 23.1 25.28 ± 16.21
Range of motion	
Flexion	**90 (85, 100)**
Extension	**0 (0, 0)**
Abduction	**30 (20, 30)**
Adduction	**20 (10, 20)**
Internal rotation	**10 (7.5, 20)**
External rotation	**0 (0, 12.5)**
VAS at rest	**3 (2, 4)**
VAS in motion	**6 (5, 7)**
Oxford Hip Score (OHS)	**20.3 ± 7.1**
Forgotten Joint Score	**14.6 (8.3, 27.3)**

Data presented as mean ± standard deviation or median (quartile 1, quartile 3) based on the normality assessment for continuous variables

*HOOS* Hip Disability and Osteoarthritis Outcome Score

**Table 2:** Patient-reported outcome measures and range of motion amongst patients undergoing robotic arm–assisted THA with functional cup positioning at 1 year post-operatively

Variables	Robotic arm–assisted THA (*N* = 30)	*P*-value*
HOOS (%) HOOS pain (%) HOOS symptoms (%) HOOS activity of daily living (%) HOOS sport and recreation function (%) HOOS hip-related quality of life (%)	97.5 (79.4, 100) 97.5 (81.25, 100) 95 (80, 100) 98.5 (86, 100) 93.8 (56.3, 100) 93.8 (71.9, 100)	0.001 0.001 ** < **0.001 0.001 0.001 ** < **0.001
Range of motion		
Flexion	100 (95, 100)	0.019
Extension	0 (0, 0)	0.317
Abduction	35 (30, 40)	** < **0.001
Adduction	30 (20, 30)	** < **0.001
Internal rotation	30 (20, 30)	** < **0.001
External rotation	20 (15, 20)	** <** 0.001
VAS at rest	0 (0, 0)	** <** 0.001
VAS in motion	0 (0, 1.5)	** <** 0.001
Oxford Hip Score (OHS)	47 (38.5, 48)	** < **0.001
Forgotten Joint Score	93.8 (53.4, 100)	** < **0.001

Data presented as mean ± standard deviation or median (quartile 1, quartile 3) based on the normality assessment for continuous variables

*HOOS* Hip Disability and Osteoarthritis Outcome Score

^*^Related samples Wilcoxon signed-rank test

**Fig. 10:**
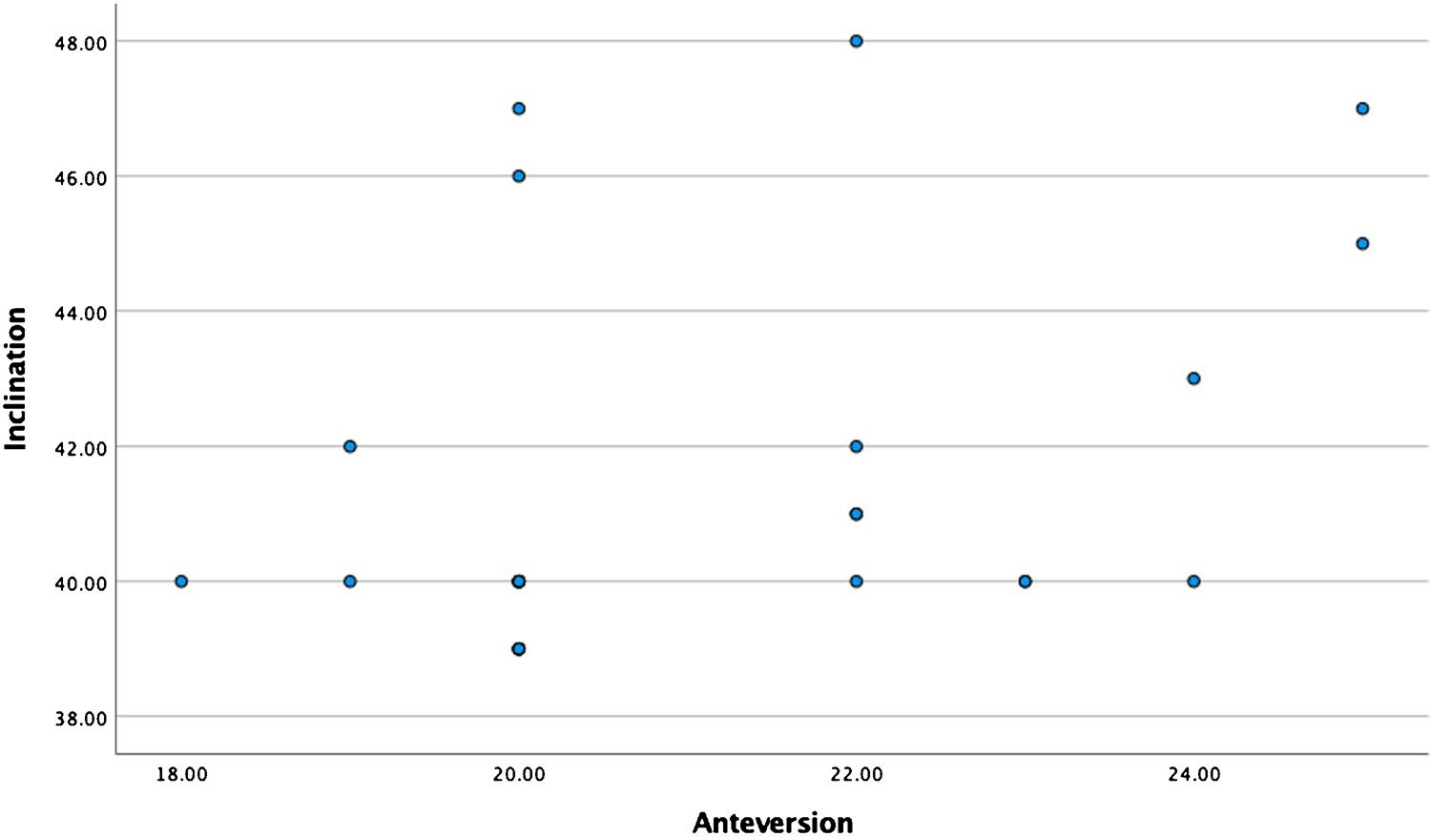
Scatter plot demonstrating the orientation of the acetabular cup in our cohort, employing a functional plan

Radiological outcomes are presented in Table 3 and Fig. 10. Mean inclination was 41.3 ± 2.7, mean anteversion 21.1 ± 1.8 and mean combined version 38.5 ± 10.8. Further interrogation of our cohort revealed that three patients had ΔSS < 10°, hence signifying spinopelvic imbalance (Study ID 17, 21, 22). In these patients, the respective functional cup inclination and anteversion were 46 and 20°, 47 and 25°, and 43 and 24°.

**Table 3:** Radiological parameters in patients undergoing robotic arm–assisted THA with functional cup positioning

Variables	Robotic arm–assisted THA
Inclination	41.3 ± 2.7
Anteversion	21.1 ± 1.8
Combined version	38.5 ± 10.8

Data presented as mean ± standard deviation

There were no dislocations at one year of follow-up. One patient experienced quadriceps weakness post-operatively and had a slow recovery, while another patient sustained a peri-prosthetic fracture at six months post-operatively following a fall, which was treated with open reduction and internal fixation.

## Discussion

The single most important surgical objective in THA is accurate implant positioning to maximise range of motion and avoid impingement. The spine-pelvis-hip construct should be regarded as a single unit that adapts during postural changes. The movements of the individual components of this construct are interwoven. For example, in patients with a stiff spinopelvic construct, loss of the posterior rollback mandates additional hip flexion in sitting position and more extension in standing, which conceptually increases the risk of impingement.

Gaining an in-depth understanding of the necessary parameters to evaluate spinopelvic mobility and recognising the individual patterns of imbalance and ramifications is vital for the arthroplasty surgeon. Robotic arm–assisted THA offers a pragmatic and reproducible way to achieve the desired implant orientation, in addition to offset and leg length [32]—all very important surgical targets especially in the subset of patients with spinopelvic imbalance. Furthermore, the introduction of the latest software allowing virtual ROM is a valuable tool in the surgical armamentarium, providing real-time pre-operative and intra-operative feedback regarding ROM and the presence of impingement.

Utilisation of dual mobility cups could also represent a practical solution in patients with a stiff spine and a bail-out option in cases where persistent impingement is evident [33, 34]. It has been shown that they can minimise dislocations after primary THA [35] and a recent study from the Australian Orthopaedic Association National Joint Replacement Registry (AOANJRR) has reported 99.1% survival at 14 months [36]. While dual mobility articulations could help mitigate the dislocation risk, one should consider that a real benefit regarding ROM is seen with the larger acetabular cups. Furthermore, the long-term survivorship of this construct in younger patients with spinopelvic imbalance needs to be substantiated whereas another consideration is the potential for fretting and corrosion between the titanium shell and cobalt-chromium liner [33, 37].

There is mounting evidence to debunk the perception that impingement and dislocations are rare if the acetabular cup is positioned within the historical safe zones [19]. Hence, it is imperative to acknowledge that no universal target can be applied to all patients and functional zones and targets should be considered individually.

Previous studies have reported clinical outcomes following individualised cup positioning. Vigdorchik et al., in their paper validating the international Hip-Spine Classification [18], reported 99.2% dislocation-free survivorship at five years’ follow-up with a dislocation rate of 0.8%.

Sharma et al. also conducted a prospective study on 1500 patients comparing the Lewinnek safe zone with novel dynamic acetabular cup planning [38]. Authors reported a very low dislocation rate 0.4% and noted that only 56% of dynamically planned cups were within the Lewinnek zone [38].

Our study is the first one to report PROMs with functional cup positioning. We noted excellent outcomes at one year across all measured scores and significant improvement compared to baseline. Furthermore, we found that patient-specific component positioning was significantly affected by the individual spinopelvic motion.

The strengths of our study include the prospective capture of data, standardisation of implants and surgical technique to avoid introducing confounders, and use of FJS that has been shown to have a higher ceiling effect [39] and suggested it could overcome some of the weaknesses of traditional PROMs. Limitations of our study are primarily owing to its small sample size, increasing the chance of type I error. Furthermore, we did not employ a comparator group and report short-term results.

Validating these signals in a larger population and investigating longer-term clinical data and dislocation rate will be a key part of substantiating the potential benefits of robotic arm assistance and functional cup positioning.
